# The immune-microbiome axis in salt-sensitive hypertension: a focus on renal and neural mechanisms

**DOI:** 10.3389/fphys.2025.1653387

**Published:** 2025-10-08

**Authors:** Leonardo Máximo Cardoso, Richard David Wainford

**Affiliations:** ^1^ Department of Biological Sciences, Institute of Exact and Biological Sciences, Federal University of Ouro Preto, Ouro Preto, Brazil; ^2^ Department of Cardiology, School of Medicine, Emory University, Atlanta, GA, United States

**Keywords:** hypertension, salt, inflammation, microbiome, short chain fatty acids, brain, kidney, salt sensitivity

## Abstract

Systemic arterial hypertension (SAH) is a prevalent condition affecting humans and other mammals, with high salt intake recognized as a major risk factor for its development and progression. This review examines the intricate interplay between dietary salt, immune signaling, neural regulation and renal mechanisms in the pathophysiology of salt-sensitive hypertension (SSH). High salt consumption not only directly influences blood pressure but also induces low-grade inflammation by activating both innate and adaptive immune responses, particularly promoting pro-inflammatory T cell (T_H_17/IL-17) and macrophage phenotypes. These immune alterations impact key organs involved in blood pressure regulation, including the kidneys and central nervous system (CNS). In the CNS, salt-induced immune activation, especially microglial activation and cytokine production in regions such as the paraventricular nucleus, enhances sympathetic outflow and contributes to neurogenic hypertension. Disruption of the blood-brain barrier further facilitates immune cell infiltration and perpetuates neuroinflammation. Additionally, recent evidence shows that high salt intake alters the gut microbiome, reducing its diversity and favoring pro-inflammatory bacterial populations, which further amplify immune dysregulation via the gut-grain axis. The role of the kidneys in sodium handling is modulated by immune cell infiltration and cytokine-drive changes in sodium channel expression, reinforcing salt sensitivity. Aging and sex differences further modulate these pathways, increasing SSH risk in older individuals and postmenopausal women. Emerging therapeutic strategies targeting the gut microbiota, immune signaling, and neural pathways offer promise improvement for SAH management. However, further research is needed to clarify causal mechanisms and optimize interventions that address the neural-immune-microbiome axis in hypertension.

## 1 Introduction

Systemic arterial hypertension (SAH), characterized by a persistent elevation in blood pressure (BP), is a clinical condition that transcends the human species, affecting various mammalian species. Hypertension is the single largest risk factor for numerous diseases, including ischemic brain infarction, intracranial hemorrhage, myocardial infarction, renal diseases, and congestive heart failure (APA, 2020; WHO, 2012; Frame and Wainford, 2017). It has been widely documented that intrinsic factors like genetic background, sex and age (Nist et al., 2024; Kim et al., 2024; Demirci et al., 2025) as well as extrinsic factor like smoking (Groppelli et al., 1992), alcohol (Lima et al., 1999), sedentarism (Alvarez et al., 1999), and eating habits (Yamamoto-Kimura et al., 1996) are associated with the development of SAH. Among eating habits, salt intake plays a key role in the development of SAH (Grillo et al., 2019) and will be further discussed here.

High salt intake can fulfill physiological and homeostatic functions, particularly when the individual sodium balance is low and body sodium content must be replenished. Under this condition, the high salt intake is driven by an increase in sodium appetite, which results in a behavioral state in which the individual seeks and consumes higher than normal salt amounts, also called *need-induced* sodium appetite (Geerling and Loewy, 2008). Curiously, mammals also exhibit high sodium intake behavior which is not driven by physiological needs for body sodium replenishment but for what is understood as a hedonic stimulus termed *need-free* sodium appetite (Geerling and Loewy, 2008). For the purpose of this review, we will focus on the *need-free* sodium intake and its impact on the BP regulation and contributions to hypertension.

High salt intake has long been associated with “stiffen and harden pulse”, a condition first described by Chinese physician Huang Di´s *Neijing Swen*, about 2500 BCE (Bailey and Dhaun, 2024; Ha, 2014). However, it was only in the beginning of the 20th century that the association between high salt intake and hypertension was established by Ambard and Beaujard using modern scientific methodology and standards (Ambard and Beaujard, 1904). Since then, several experimental and trial studies have supported these observations and provided evidence for the impact that high salt intake has on the pathophysiology of hypertension. In the 1940´s and 1950´s, studies by Kempner showed that the BP in 2 of the 6 patients under a strict rice fruit diet declined to normal levels and rose again after 20 g of sodium chloride was added daily (Grollman et al., 1945). In essence, this observation gave birth to the idea that would be later elaborated into the concept of the salt-sensitivity of BP (SSBP). The SSBP can be currently defined as a trait where the individual BP (animal or human) displays a change parallel to the changes in salt intake (Eli et al., 2016; Weinberger, 1996a; Weinberger, 1996b; Weinberger, 2006). The SSBP is a trait normally distributed in humans and is present in both the normotensive and hypertensive individuals. An important and influential American study showed that after salt depletion and following sodium load, 26% of the *normotensive* patients were ascribed as salt sensitive while 51% of the *hypertensive* patients were salt sensitive (Weinberger et al., 1986). Despite limitations (Bailey and Dhaun, 2024) this study established that hypertensive patients have higher probability of displaying a SSBP phenotype and launched the basis for the understanding that salt sensitivity is an important cardiovascular risk factor “independent of and as powerful as BP” by itself (Bailey and Dhaun, 2024; Eli et al., 2016).

Over the last 100 years scientific investigations have repeatedly demonstrated that renal pressure-natriuresis, hormonal hydroelectrolytic balance and neural sympathetic mediated events are key to the SSBP (Bie and Evans, 2017a; Bie and Evans, 2017b). These studies have revealed intricate interactions among different systems to sustain high BP and other detrimental outcomes of high salt intake. Of particular interest in this context is a growing body of evidence that has linked the immune cellular and signaling mechanisms not only to hypertension but also to the SSBP (Bailey and Dhaun, 2024; Mattson, 2014; Mattson, 2019; Rucker et al., 2018; Maaliki et al., 2022) and experimental and clinical studies led to the idea that hypertension may be an “inflammatory” disease (Boos and Lip, 2006; Solak et al., 2016). Although the immune events that take place along hypertension development and maintenance hold some characteristics of an “inflammatory state” such as heightened immune-related signaling molecules in end-organs linked to BP control and hypertension, it is crucial to acknowledge that this concept may deviate from the conventional definition of inflammation. Classically, inflammation is defined as a protective process by which the body’s immune system responds to injury, infection, or harmful stimuli. The four typical signs of acute inflammation were first described by Celsus in ancient Rome (30–38 B.C.) (Chandrasoma and Taylor, 1998) and are defined as: Rubor (redness), Calor (heat), Tumor (swelling) and Dolor (pain). Those signs are not always present in end-organs associated with the development of hypertension and make one wonder whether “inflammation” truthy describes the role of the immune system in the development of hypertension. However, some authors have elaborated a new concept to describe a chronic, subclinical, sterile and systemic condition characterized by a persistent, mild elevation of inflammatory markers in the body (Minihane et al., 2015; Ronnback and Hansson, 2019; van de Vyver, 2023) that would better represent the role of the immune system in hypertension. This concept is termed “low-grade inflammation” (LGI) or “metabolic inflammation” or “metainflammation” (van de Vyver, 2023) and has been implicated in the pathogenesis of many non-communicable chronic diseases, including salt-sensitive hypertension (SSH) (Boos and Lip, 2006; Al-Nimer and Alhusseiny, 2016).

Based on the currently available experimental evidence, this review will explore some aspects of the intricate interplay between the immune system and its pro-inflammatory signaling molecules (PIM), the central nervous system (CNS) and the renal system in SSH.

## 2 The immune system and the salt-sensitivity of blood pressure: an overview

The role of the immune system in BP regulation and hypertension development became evident with pharmacological studies in rodents showing that, under certain conditions, the inhibition or lacking of cyclooxygenase-1, a key enzyme in the prostanoids synthesis pathway, can lower the BP of Ang II-salt hypertensive rats (Asirvatham-Jeyaraj et al., 2013; Athirakul et al., 2001). In addition, a 2007 study by Guzik and cols. Showed that activation of immune cells contributed to the hypertension induced by angiotensin II (Ang II) infusion and deoxycorticosterone acetate (DOCA) plus high-salt diet in *Rag1* ^−/−^ mutant mouse (Guzik et al., 2007). This particular study showed that the knockout mice for *Rag1* lack mature T cells and B cells, which protected them from vascular dysfunction and remodeling in addition to reducing the hypertensive responses to Ang II and DOCA-salt (Guzik et al., 2007). Clinical studies also showed that patients with hypertension had a higher proportion of immunosenescent, proinflammatory, and cytotoxic CD8^+^ T cells in their blood. Furthermore, immunohistochemical assessment of renal biopsy specimens from patients with hypertensive nephrosclerosis revealed increased expression of the T-cell chemokine interferon (IFN)-inducible T-cell α chemoattractant in the proximal and distal tubules suggesting that T-cell-driven inflammation may also play a role in human hypertension, especially in the kidney (Youn et al., 2013).

One of the most important risk factors for hypertension, high salt intake, is found to alter the activation of inflammatory cells in both the innate and adaptive immune system (Afsar et al., 2018; Miyauchi et al., 2024; Li et al., 2022). Murine macrophages and T cells exposed to high salt have been found to express a pro-inflammatory state both, *in vitro* and *in vivo* (Binger et al., 2015; Jantsch et al., 2015; Kleinewietfeld et al., 2013; Wu et al., 2013; Zhang WC. et al., 2015). Additionally, a modest increase in extracellular salt concentration induces the serum glucocorticoid kinase 1 (SGK1) expression, a serine/threonine kinase 4 that promotes IL-23R expression by deactivating the transcription factor Forkhead box protein O1 (FOXO1) in mice. FOXO1 is a direct repressor of IL-23R expression and enhances the IL-17-producing T helper lymphocyte cells (T_H_17) differentiation *in vitro* and *in vivo* through IL-23 mechanisms (Wu et al., 2013). The T_H_17 polarization of *naïve* immune cells has been reported in both humans and mice (Miyauchi et al., 2024; Kleinewietfeld et al., 2013; Jorg et al., 2016; Wilck et al., 2017; Yakoub et al., 2024). Also, high salt concentrations in the medium can increase the production of PIM like TNF-α, IL-2 and granulocyte-macrophage colony-stimulating factor by cultured T_H_17 cells (Kleinewietfeld et al., 2013). Moreover, the SGK1 activation has also been shown to stimulate the mineralocorticoid-mediated expression of epithelial sodium channels (ENaC) in the kidneys of mice (Wulff et al., 2002) what indicates that the SGK1 pathway is shared by inflammatory and sodium homeostasis mechanisms.

High salt also impacts macrophage core functions both *in vitro* and *in vivo*, especially because macrophages exhibit chemotactic migration in response to salt gradients *in vitro* (Muller et al., 2024). The incubation of bone marrow-derived macrophages with high concentrations of sodium chloride (NaCl) elicited a strong pro-inflammatory phenotype characterized by enhanced pro-inflammatory cytokines (PIC) production, increased expression of immune-stimulatory molecules, and an antigen-independent boost of T cell proliferation through pathways that may involve the nuclear factor kappa-light-chain-enhancer of activated B cells (NF-κB) and the mitogen-activated protein kinase (MAPK) signaling (Hucke et al., 2016). Additional evidence shows a direct action of NaCl in inducing pro-inflammatory phenotypes. Zhang and cols. Showed that a roughly 33% increase in medium NaCl concentration significantly induced interleukin 6 (IL-6) and MCP-1 (or CCL2) production by retinal pigment epithelium (ARPE-19) cells in culture (Zhang D. et al., 2015). This effect was not mediated by osmolarity, as an equivalent osmotic mannitol addition to the medium had no effect on IL-6 or MCP-1 production (Zhang D. et al., 2015). In addition to the NF-κB and MAPK pathways, evidence from humans and experimental animal studies have shown that myeloid-specific janus kinase 2 (JAK2) also contributes to LGI and SSH. The JAK2 signaling pathway plays crucial roles in many cellular processes and physiological functions such as cell growth and survival, hematopoiesis, immune regulation, inflammation, metabolism and gene transcription (Bader and Meyer, 2022). High salt upregulated gene expression of the JAK/STAT/SMAD pathway in human monocytes and the ablation of JAK2 signaling attenuated the SSH hypertension in mice (Saleem et al., 2024). The authors also found that JAK2 increased production of highly reactive isolevuglandins (IsoLG) and IL-6 by renal antigen presenting cells (APC). In addition, JAK2 also activates T cells and increases production of IL-6, interleukin 17A (IL-17A), and tumor necrosis factor-alpha (TNF-α) in mice (Saleem et al., 2024).

Although the evidence suggests that sodium can drive inflammation-related signaling pathways in immune cells, increased salt intake actually produces very small Cohen effect size in steady-state plasma sodium concentrations of certain models of SSH, such as the Dahl-salt-sensitive model (DSS) (Stocker, 2023; Nakamura and Cowley, 1989) or the HS12W model (Gomes et al., 2017). This poses some limitation to the general idea that high salt intake can affect immune cell physiology. It also may suggest that the activation of the immune system by high salt intake may include an indirect result of increased sodium “traffic” throughout the body. Indeed, assessment of serum sodium concentrations in humans showed only a modest (∼1.4%) increase in systemic serum sodium concentration after a high salt meal, lasting at least 2 h, and without changes in hormones involved with body fluid homeostasis or endothelial function, such as endothelin-1 (ET-1), vasopressin (AVP), and atrial natriuretic peptide (ANP) (Dickinson et al., 2014). One important question here yet to be answered is whether this transient increase in sodium concentration of the extracellular fluid could be enough to unleash important changes in immune cells phenotype toward a pro-inflammatory state and contribute to the LGI over time? Since sodium absorption by the intestine doesn´t distribute throughout body volume at once, the great gradient of sodium through the intestine wall could make these local immune cells more susceptible to sodium-driven changes in phenotype and cytokines profile production as described in *in vitro* studies. On the other hand, recent evidence has shown that high salt intake can result in non-osmotic sodium accumulation in the skin (Titze et al., 2003) and muscle tissue (Rossitto et al., 2020) pointing toward the possibility that immune cells in these regions could also be impacted by high sodium gradients and contribute to systemic LGI.

In addition to salt-induced immune cell differentiation and activation, sodium can enter into dendritic cells (DC) via the amiloride-sensitive epithelial sodium channel (ENaC) and activate the production of the PIM IL-1β and promote T cell production of IL-17A and IFN-γ (Barbaro et al., 2017). It has been proposed that higher sodium influx through ENaC in DC increases sodium/calcium exchange, thus activating phosphokinase C (PKC) and NADPH-oxidase (Barbaro et al., 2017). The increased reactive oxygen species production drives the production of IsoLG and the NOD-like receptor family pyrin domain containing 3 (NLRP3) inflammasome that activates caspase-1 and increases the production of IL-1β by the DC (Pitzer et al., 2022). Interesting, when such activated DC are adoptively transferred into *naïve* mice, they prime hypertension in response to a sub-pressor dose of Ang II (Barbaro et al., 2017). These experimental findings are further supported by a study showing that amiloride exerts anti-inflammatory effects by decreasing the PMI TNF and IL-6 plasma levels, but not IL-17A levels, in patients with resistant hypertension and type 2 diabetes (Thangaraj et al., 2024).

From a vascular perspective, transient high salt intake promotes T-cell-mediated hypertensive vascular injury in a mice model of high-salt intake drinking 1% NaCl solution (Yakoub et al., 2024). The authors showed that in transient high salt-treated hypertensive mice, the aortic injury was associated with increased inflammation, accumulation of neutrophils, monocytes, CD69^+^CD4^+^ T cells, as well as CD4^+^ and CD8^+^ memory T cells. Moreover, high salt intake sensitized the animals for angiotensin II-induced vascular lesions by increasing the expression of aortic RORγt as well as splenic CD4^+^T_H_17 and CD8^+^TC1 T cells in Ang II-treated mice (Yakoub et al., 2024). The authors suggested that transient high salt intake induces a subclinical T-cell-mediated aortic immune response, which is enhanced by Ang II and contributes to the vascular lesion in these animals.

Collectively, these data strongly indicate that sodium can deeply affect the physiology of immune cells toward a LGI state, which strongly correlates with SSH. Certain pieces of evidence suggest that sodium-induced modifications of immune cells physiology have a causal relationship with hypertension and further studies are needed to fully understand the role of the immune signaling system in the pathophysiology of hypertension.

### 2.1 Key points

1. The immune system influences BP regulation and the development of hypertension.
2. High salt intake triggers a pro-inflammatory state in both innate and adaptive immune systems affecting macrophages and T cells and promoting the production of pro-inflammatory mediators.
3. Sodium can modify macrophage physiology leading to pro-inflammatory phenotypes, increased production of PIM (like IL-6 and MCP-1), immune-stimulatory molecules, and activation of NF-κB and MAPK signaling pathways.
4. Sodium influx through ENaC in dendritic cells can induce production of IL-1β through inflammasome activation and promote the IL-17A and INF-γ production by T cells.

## 3 Salt-sensitive hypertension and the central nervous system immune system

The central nervous system (CNS) plays a pivotal role in the BP regulation, both in normotension and hypertension (Guyenet, 2006) and this concept has been grounded on several findings in the literature showing that neurogenic mechanisms are important for heightened BP and, especially, for SSH (Eli et al., 2016; Maaliki et al., 2022; Adams et al., 2008). The major findings in this section are summarized in Table 1.

**TABLE 1:** Known effects of the immune system on neurogenic blood pressure control that contribute to salt sensitive hypertension and the outcomes of interventions.

Hypertension model	Type of immune response	Target (Site/Cell)	Major PIM involved	Effects on target	Mechanistic insights	Intervention outcomes	Refs
Dahl salt-sensitive rats (DSS)	Cellular and Humoral	PVN microglia, astrocytes, perivascular macrophages	IL-1β, IL-6, TNF-α, IL-17	• Increased microglial activation, oxidative stress and cytokine production in PVN • Reduced baroreflex sensitivity	Increased immune signaling in the PVN resulting in increased sympathetic activation	Minocycline or IL-1β receptor inhibition in PVN attenuates BP, sympathetic nerve activity, oxidative stress and cytokine imbalance	Yu et al. (2022), Qi et al. (2016), Jiang et al. (2018), Moreira et al. (2019), Gao et al. (2022)
High salt diet (Sprague-Dawley rats, mice)	Cellular and Humoral	PVN microglia, astrocytes	TNF-α IL-1β, IL-6	• Increased microglial and astrocyte activation • Increased cytokine production • Oxidative stress	Increased immune signaling in the PVN resulting in increased neuronal activation Reduced inhibitory neuronal activity through Gαi2 protein	Minocycline or Gαi2 protein preservation prevents microglial activation and hypertension	Moreira et al. (2019), Liu et al. (2022), Gilman et al. (2019)
DOCA-salt treated mice	Cellular and Humoral	CNS (PVN), perivascular macrophages	IL-1β	• Increased IL-1β signaling in PVN • Sympathetic activation	Increased sympathetic activity due to higher IL-1β stimulation in the PVN.	IL-1R knockout or antagonism (anakinra) reduces neurogenic pressor activity and BP	Baumann et al. (2024)
High salt + experimental autoimmune encephalomyelitis (EAE, mice)	Cellular and Humoral	CNS endothelial cells, microglia, astrocytes	IL-17, GM-CSF, TNF-α, IL-2	• BBB disruption • Enhanced leukocyte infiltration • Loss of tight junctions • ROS production • Microglial and astrocyte activation	Increased BBB disruption with increased T_H_17 infiltration in the brain tissue	IL-17A neutralizing antibodies or ROS/myosin light chain pathway blockade prevent BBB disruption	Huppert et al. (2010)
High salt intake (general, huma/animal)	Humoral	CNS, kidney, vascular endothelium	IL-6, IL-17	• Increased IL-6 expression in kidney and CNS • Endothelial dysfunction • Immune cell infiltration	Increased IL-6 expression leading to low-grade inflammation	IL-6 antibody attenuates hypertension, albuminuria, and renal injury in Dahl salt rats	Sesso et al. (2007), Chamarthi et al. (2011), Hashmat et al. (2016)
Spontaneously hypertensive rats (SHR)	Cellular and Humoral	PVN microglia, CNS-infiltrating immune cells	TNF-α, IL-1β	• Increased central and peripheral inflammation • Microglial activation in PVN.	Increased infiltration of immune cells into the CNS leading to low-grade inflammation and sympathoexcitation	Minocycline or bone marrow replacement with WKY reduces BP and inflammation TLR4/AT1R inhibition reduces BP and cytokines in RVLM/NTS.	Santisteban et al. (2015)
Ang II or l-NAME-induced hypertension	Cellular and Humoral	CNS (PVN, RVLM, NTS), perivascular macrophages	IL-17, TNF-α, IL-1β	• Increased BBB permeability • Perivascular macrophage activation • ROS production • Neurovascular dysfunction	Increased infiltration of immune cells into the CNS leading to low-grade inflammation and sympathoexcitation	IL-17 knockout prevents hypertension; microglia ablation reduces BP and cytokine expression in PVN.	Biancardi et al. (2014), Faraco et al. (2016), Shen et al. (2015), Madhur et al. (2010)

PVN, paraventricular nucleus of the hypothalamus; IL, interleukin; TNF, tumoral necrosis factor; BP, blood pressure; DOCA, deoxycorticosterone acetate; CNS, central nervous system; IL-1R, interleukin 1 receptor; GM-CSF, granulocyte-macrophage colony-stimulating factor; ROS, reactive oxygen species; BBB, blood brain barrier; TLR4, Toll-like receptor 4; AT1R, angiotensin II, receptor 1; RVLM, rostral ventrolateral medulla; NTS, nucleus tractus solitarius; l-NAME, l-N^G^-nitro-l-arginine methyl ester.

The reflex control of BP regulation has been widely recognized for its ability to short-term buffer large BP fluctuations and prevent potentially harmful sudden increases or decreases in systemic BP (Guyenet, 2006; Wehrwein and Joyner, 2013). In SSH, salt-dependent increases in BP are usually accompanied by reduced baroreflex sensitivity (Ozaykan et al., 2017; Rosa et al., 2020; Wang et al., 2005). In addition to salt-related baroreflex impairments, excessive salt intake also leads to impairment in the reflex response to cardiopulmonary reflex activation. These reflexes are volume/chemical sensing mechanisms that regulate sympathetic and parasympathetic outflow to the cardiovascular system in response to volume expansion and/or chemical activation with metabolic byproducts and neurochemicals (Vasquez et al., 1997; Vasquez, 1994). In Dahl salt rats, a high salt diet sensitized the cardiopulmonary reflex-driven sympathetic response to hypervolemia in Dahl salt resistant rats but not in Dahl salt sensitive rats (Victor et al., 1986). Interestingly, Dahl salt sensitive rats had a smaller sympathetic inhibition response when challenged with volume expansion compared to Dahl salt resistant rats, even under low-salt diet (Ferrari A. et al., 1984; Ferrari AU. et al., 1984). These findings suggest that the salt-sensitivity trait by itself includes a reduced sensitivity of the cardiopulmonary reflex in Dahl salt sensitive rats regardless of high salt intake. Since these studies were conducted in urethane-anesthetized rats, the full understanding of the cardiopulmonary reflex role in SSH regulation remains limited. However, it provides important evidence of changes in short-term BP regulation due to the salt-sensitive trait. The plasticity of the cardiopulmonary reflex has also been investigated in hypertensive humans and, consistent with experimental findings, the results revealed that high salt intake potentiates the cardiopulmonary reflex gain and atrial natriuretic factor response only in salt resistant hypertensive patients (Trimarco et al., 1991). Interestingly, the reflex response to carotid baroreceptor unloading was unaffected by salt loading in none of the groups (Trimarco et al., 1991) suggesting that cardiopulmonary reflexes are more sensitive than baroreflex to high salt intake in salt resistant hypertensive patients.

The paraventricular nucleus of the hypothalamus (PVN) is a hypothalamic nucleus that orchestrates neural and hormonal responses to changes in blood sodium concentration, osmolality and BP through two distinct cell subtypes: the parvocellular neurons, which comprise autonomic regulatory neurons (Ferguson et al., 2008; Osborn et al., 2007), and the magnocellular neurons, which include oxytocin and vasopressin producing/containing neurons (Antunes-Rodrigues et al., 2004). Therefore, the PVN is a key forebrain region that plays important roles in neurogenic hypertension (Osborn et al., 2007). While the majority of descending sympathetic-related projections originating from PVN parvocellular neurons connect to the rostral ventral lateral medulla (RVLM) in the brainstem or to the intermedial lateral column in the spinal cord (Guyenet, 2006; Dampney et al., 2018), the majority of the projections from magnocellular PVN neurons project to the neurohypophysis and release vasopressin and oxytocin to the pituitary portal system when activated (Antunes-Rodrigues et al., 2004). The outcome is a multifaceted role of the PVN in regulating BP levels and sodium excretion by the kidney which contributes to the pathophysiology of SSH.

The immune signaling mechanisms in hypothalamic and brainstem regions controlling BP have received considerable attention in the recent years and the evidence gathered so far point toward important immune influence on the functional role of these regions in the SSH. The blockade of microglia activation with minocycline, a selective CNS-acting non-steroidal anti-inflammatory drug, in Dahl salt-sensitive rats can inhibit the augmented local production of IL-1β, IL-6, TNF-α and the oxidative stress in the PVN. Likewise, minocycline infusion in the PVN also attenuated the high BP, the ratio between resting RSNA/MaxRSNA, central prostaglandin E_2_ and plasm norepinephrine levels (Yu et al., 2022). The infusion of the IL-1β receptor inhibitor gevokizumab into the PVN of Dahl salt-sensitive rats also attenuated the BP, heart rate, and plasma norepinephrine levels (Qi et al., 2016) suggesting that this specific cytokine plays a role in the PVN-driven neurogenic pressor response. The authors also reported increased levels of NOX-2, NOX-4, IL-1β, NLRP3, Fra-LI and lower levels of IL-10 in Dahl salt-sensitive rats fed high salt diet and that gevokizumab restored the balance in the PVN (Qi et al., 2016). In addition, Jiang and cols. (2018) reported that Dahl salt-sensitive rats under high salt diet exhibited higher expression of PIC like TNF-α, IL-6, IL-1β and Fra1in the PVN and that intracerebroventricular infusion of highs sodium solution produced a marked increase in the expression of TNF-α, IL-6, IL-1β in Sprague-Dawley rats (Jiang et al., 2018). These studies highlight the role of high sodium concentrations in the PVN and its correlation to increased PIC production as well as the effect of the local anti-inflammatory agent minocycline or the IL-1β receptor inhibitor gevokizumab on SSH.

Although extensive research has advanced our understanding of immune signaling in the PVN regarding SSH, the role of immune signaling in the brainstem and its relationship to SSH is less advanced. It is known, however, that physiological reflex activation of the sympathetic circuit by bilateral carotid occlusion decreases the plasma levels of TNF and IL-1β, and increased the levels of IL-10 in endotoxemia induced by lipopolysaccharide in rats (Brognara et al., 2021a). Likewise, the activation of the Bezold-Jarisch reflex (the chemosensitive cardiopulmonary reflex) in endotoxemic rats reduced plasma levels of TNF and spleen levels of IL-6 (Brognara et al., 2021b). Although this evidence suggests that the autonomic activation of descending vagal pathways may influence peripheral PIM production, it does not provide further insights on how PIM can influence reflex regulation of the BP in the brainstem. On the other hand, toll-like receptor 4 and angiotensin II receptor 1 inhibition reduces BP as well as IL-6 and TNF-α protein density in the RVLM and nucleus tractus solitarius (NTS) of spontaneous hypertensive rats corroborating the idea that immune signaling in neuronal circuitries controlling BP is important for the hypertension development (Mowry et al., 2021).

### 3.1 Peripheral immune cell infiltration in the CNS and hypertension

Peripheral immune cell infiltration in the CNS has been associated with enhanced sympathetic drive, resulting in BP increase, whereas inhibition of this immune signaling in the CNS ameliorates hypertension (Nist et al., 2024; Moreira et al., 2019; Xue et al., 2016; Yu et al., 2010). This has been demonstrated by a study in which chimeric spontaneously hypertensive rats (SHR) reconstituted with Wistar-Kyoto (WKY) bone marrow resulted in significant BP reduction associated with attenuation of both central and peripheral immune activation (Santisteban et al., 2015). The authors also reported elevated BP and increased central and peripheral “inflammation” in chimeric WKY rats reconstituted with SHR bone marrow (Santisteban et al., 2015). When microglia activation was pharmacologically inhibited with minocycline, hypertension was attenuated in SHR (Santisteban et al., 2015). These findings strongly suggest that extravasation of bone-marrow-derived cells into the CNS, particularly into the PVN, is an important event in hypertension.

The infiltration of circulating immune cells into the CNS is believed to be associated with increased permeability of the blood-brain barrier (BBB) (Dinh et al., 2025). Hypertension has been shown to be linked to increased BBB permeability in the PVN, NTS and RVLM regions (Nist et al., 2024; Biancardi et al., 2014; Ueno et al., 2004). However, a causal relation between increased BBB permeability and hypertension is yet to be determined. Such an increase in the BBB permeability has been shown to allow systemic Ang II to enter into the nervous tissue parenchyma thus activating AT_1_ receptor on perivascular macrophages (PVM) within the brain (Biancardi et al., 2014; Faraco et al., 2016). The activation of PVM promotes pathogenic actions in key cardiovascular controlling regions of the brain to set neurovascular dysfunction through reactive oxygen species (ROS) production via the superoxide producing enzyme NOX_2_ during chronic hypertension (Faraco et al., 2016). Interestingly, the autonomic nervous system (SNA) also innervates bone marrow (Katayama et al., 2006) and spleen (Carnevale et al., 2016), and increased sympathetic outflow can stimulate mobilization and release of hematopoietic stem cells into blood stream through adrenergic neurotransmission stimulation (Hanoun et al., 2015; Sant et al., 2016). Sympathetic stimulation of bone marrow has been shown to favor enhanced proinflammatory responses in a mature innate immune system (Harwani et al., 2012). Selective ablation of splenic nerve prevents T cell egression from spleen and infiltration into renal and aorta tissue and protects against hypertension (Carnevale et al., 2016). This interplay between sympathetic drive and peripheral immune cell stimulation has the potential to contribute to a positive feedback loop in which more peripheral cells can infiltrate the CNS and stimulate local immune signaling allowing further sympathetic stimulation and, ultimately, aggravates the hypertension over time. However, increased sympathetic drive is a common finding in individuals with hypertension already established and raises the question of whether hypertension-related LGI is cause or consequence of high BP. For instance, unilateral renal sympathetic denervation, an approach which reduces BP, diminishes inflammatory cells activation in experimental models of hypertension (Xiao et al., 2015) and in human hypertensive patients (Zaldivia et al., 2017). One limitation here is that clinical studies could not discriminate between direct and indirect outcomes from renal denervation regarding direct nerve ablation effects on BP *versus* indirect effect resulting from attenuation of innate immunity on BP.

### 3.2 Microglia and salt-sensitive hypertension

The local production of pro- and anti-inflammatory molecules by microglia has also been reported as an important source of immune regulation of BP in SSH (Moreira et al., 2019; Shen et al., 2015; Shi et al., 2010). Increased activation of microglial has been implicated in cardiovascular disease and hypertension development. Microglial, characterized by their branched morphology, are tissue-resident macrophages of the CNS, and play pivotal role in monitoring the presence of pathogens in the CNS tissue and modulating synaptic and neuronal activities (Wang et al., 2022). Microglia activation states are based on the peripheral macrophages classification and include the M1 and M2 states in addition to the resting state M0 (Wang et al., 2022; Dubbelaar et al., 2018). Microglia activation is accompanied by morphological shifting from a small soma cell with ramified protrusions to an amoeboid-like morphology that enables microglia motility and phagocytic function (Dubbelaar et al., 2018; Kettenmann et al., 2011). This transition involves a complex interplay of different molecular and cellular mechanisms that include NF-κB signaling (Singh et al., 2022). Current literature on the molecular mechanisms leading to microglia activation in SSH primarily focuses on hypertension in a general manner and lacks specific aspects pertaining to the SSBP. In addition to the pattern recognition receptors expressed by microglial cells like the Toll-like receptors (TLR2, TLR3 TLR4 and TLR9) that can activate microglial cells (Olson and Miller, 2004), pro-inflammatory cytokines like IL-1β (Swaroop et al., 2016), IL-6 (Recasens et al., 2021), TNF-α (Bras et al., 2020), and INF-γ (Olson and Miller, 2004) can also activate microglial cells. Given the involvement of IL-1β, the factors that also activate NLRP3 inflammasome, as discussed above (Pitzer et al., 2022; Menu and Vince, 2011), may significantly contribute to microglia activation in response to high salt exposure. Interesting, the levels of specific immune signaling molecules can polarize resting microglia to the M1 or M2 states and, therefore, drive transition toward pro or anti-inflammatory phenotypes of active microglia. For instance, the signaling by LPS and INF-γ drive the microglia polarization toward the M1 phenotype which increases the expression of pro-inflammatory modulators like TNF-α, INF-γ, iNOS IL-1β, IL-2, IL-6, COX-2 CXCL9 and CXCL10 by microglia (Li et al., 2021). On the other hand, the signaling by IL-4 and IL-13 or LPS, IL-1β and TNF-α or IL-10 and TGF-β drive the polarization toward the M2 phenotype and increase the expression of mostly anti-inflammatory regulators (Li et al., 2021).

The role of microglia in hypertension has been demonstrated by a study in which selective pharmacological ablation of microglia in transgenic CD11b-DTR mice with either Ang II or L-N^G^-nitro-l-arginine methyl ester (l-NAME) induced hypertension reduced BP, attenuated the expression of TNF-α, IL-1β, and glutamate receptors in the PVN as well as the plasma levels of vasopressin, and kidney norepinephrine concentrations (Shen et al., 2015). These findings indicate that neuronal excitation of hypothalamic pathways involved with sympathetic (parvocellular cells in PVN) and hydric (vasopressin secretion) control is under influence of microglial cells and the local action of cytokines. Yet, the fact that kidney norepinephrine concentrations are also diminished in microglia-depleted animals indicate that sympathetic-mediated changes in renal function are also affected by local immune signaling within the PVN.

The mechanism by which high salt intake induces the activation of microglia in Sprague-Dawley rats may involve a Gαi2 signal transduction pathway. The downregulation of brain G*α*i2 proteins by continuous *i. c.v.* Infusion of a phosphodiesterase ODN probe that selectively and specifically targets G*α*i2 proteins not only produced a minocycline sensitive hypertension due to high salt intake but also increase the number of active microglia cells in the PVN of Sprague-Dawley rats (Moreira et al., 2019).

The activation of PVN-specific microglial cells in Sprague Dawley rats due to high salt intake (Moreira et al., 2019; Liu et al., 2022) is commonly associated with increased sympathetic-vascular coupling drive of the vascular tonus (Liu et al., 2022) and plasma noradrenaline (Moreira et al., 2019; Liu et al., 2022). In addition, high salt intake resulted in a more than double activation of microglia in the PVN of Dahl-salt-sensitive rats (DSS) and was also associated with elevated renal sympathetic nerve activity and production of central prostaglandin E_2_ as well as increased oxidative stress in the same region (Yu et al., 2022). In addition to rat models of SSH, mice under high salt diet also exhibited increased microglia activation and increased production of TNF-α in the PVN (Gilman et al., 2019). Most of these studies were associated with salt-related hypertension and indicate an important contribution of the microglia activation in the PVN to the SSH.

### 3.3 Central-acting interleukins/cytokines and salt sensitive hypertension

From a molecular perspective, several CNS-acting cytokines and interleukins, both peripherally and centrally generated, are involved with hypertension and include IL-1β, IL-6, IL-17, TNF-α and INF-γ while the major anti-inflammatory cytokine related with CNS immune signaling in hypertension is IL-10 (Yu et al., 2022; Moreira et al., 2019; Liu et al., 2022; Gilman et al., 2019; Yang et al., 2022; Konsman, 2022). The challenges imposed by high salt exposure have been shown to stimulate the production of IL-1β, IL-6, TNF-α (Afsar et al., 2018; Deng et al., 2017), IL-17 and differentiation of T_H_17 cells (Afsar et al., 2018; Balan et al., 2022), upregulation of MCP-1 and MIP-2 chemokines (Afsar et al., 2018; Yang et al., 2022), activation of microglia (Deng et al., 2017), activation of JAK2/STAT3 pathway in astrocytes (Deng et al., 2017) and reduce noninflammatory innate immune cell activation through reduction of IL-4 and IL-13-stimulated macrophage (Binger et al., 2015). The prevailing hypothesis suggests that higher production/action of immune signaling molecules in the CNS contributes to the heightened activity of neuronal pathways regulating BP through sympathetic activity and, therefore, contributes to hypertension. The immune signaling molecule production that is influenced by high salt consumption alters the immune balance within the CNS, favoring a pro-inflammatory profile. This shift in immune balance contributes to the development of sodium-induced neurogenic hypertension through brain-peripheral organs axis mechanisms, especially the gut-brain axis.

#### 3.3.1 IL-17

A recent concept has linked the immune related change that occurs in the gut as a consequence of high salt intake with immune related events that occur in the brain to produce hypertension. This concept has been referred to as the gut-brain axis and has T_H_17 cells and IL-17 as their major focus of investigation (Carabotti et al., 2015; Dai et al., 2023; Palmu et al., 2021). As reported by Wang and cols. (2012), clinical findings support the idea that T_H_17/IL-17 play a role in the development of essential hypertension in humans as a positive correlation was found between hypertensive and non-hypertensive groups and T_H_17 cells count in the peripheral blood (Wang et al., 2012). Other findings indicate that IL-17 may be associated with hypertension development because knockout mice for IL-17 do not sustain hypertension produced by chronic infusion of Ang II (Madhur et al., 2010). The authors suggest that endothelial dysfunction driven by oxidative stress is the most likely molecular mechanism involved in this process and may be triggered by IL-17 (Madhur et al., 2010). On the other hand, clinical evidence indicates that neither IL-17-producing cells (Youn et al., 2013) nor circulating IL-17 levels (Al-Nimer and Alhusseiny, 2016) are positively correlated with increased levels of BP. However, one *caveat* must be considered here: most of the clinical studies searching for a correlation between circulating levels of IL-17 and hypertension were carried out in patients with stablished hypertension, many under pharmacological treatment for hypertension and no assessment of salt intake. assessment.

IL-17 is a PIM produced by the T_H_17 subset of CD4^+^ T cells and is an important signaling molecule in both acute and chronic inflammatory processes (Iwakura et al., 2011). It has been recently implicated in SSH (Wilck et al., 2017; Aguiar et al., 2017; Wenzel et al., 2019) and, since then, it has been ascribed as an important immune-derived player in the SSH. Experimental data have shown that high-salt intake increase the intestinal gut population of T_H_17 lymphocytes (Jorg et al., 2016; Wilck et al., 2017; Wenzel et al., 2019) and, consequently, augmented IL-17-mediated signaling throughout the body, especially when occurring in a pro-inflammatory environment (Matthias et al., 2020). Among the effects of IL-17 in the brain, it has been shown to disrupt the blood-brain-barrier (Kebir et al., 2007), drive local activation of glial cells as well as local production of IL-17 by glial cells (Deng et al., 2017; Waisman et al., 2015), and activate its receptor (IL17RA/RC) on microglia and astrocyte in the CNS (Zimmermann et al., 2013). In addition to the action on glial cells, IL-17 can directly modulate neuronal synaptic transmission by enhancing excitatory postsynaptic currents and suppress inhibitory postsynaptic synaptic currents and γ-aminobutyric acid (GABA) induced currents in lamina IIo somatostatin-expressing neurons in mouse spinal cord slices (Luo et al., 2019).

T_H_17 lymphocytes are shown to promote BBB disruption and favor a pro-inflammatory state of the CNS (Kebir et al., 2007). The mechanisms seem to involve the activation of IL-17 and IL-22 receptors on brain endothelial cells, and their activation permeabilizes the BBB (Kebir et al., 2007). This contributes to the migration of other immune cells into the brain parenchyma as well as the translocation of larger molecules (immune molecules included) from the blood to the brain tissue. In the CNS, IL-17 can activate microglia and further cause microvascular dysfunctions (Zimmermann et al., 2013). The authors state that their data argues against a direct role of IL-17A in producing neuronal tissue damage but acts as a modulating factor in a network of locally produced cytokines (Zimmermann et al., 2013). This interpretation is shared by other authors showing that IL-17 recruits other inflammatory cells to the CNS in hypertension (Harrison et al., 2012). Importantly, experimental evidence that direct microinjection of IL-17 into the lateral ventricle or PVN can elicit increases in BP, heart rate and renal sympathetic nerve activity strongly indicates that IL-17 can influence neuronal pathways controlling BP (Cao et al., 2021).

Although many studies imply that high polarization of T_H_17 cells is linked to a high production of IL-17, much of the evidence is, indeed, provided by *in vitro* experiments (Kleinewietfeld et al., 2013; Wu et al., 2013; Wenzel et al., 2019) and evidence of the interplay between high IL-17 production and neurogenic mechanisms leading to SSH are still fledgling. To the best of our knowledge, literature lack solid evidence that high salt intake increases the circulating levels of IL-17, what may suggest that infiltration of IL-17-producing cells may be the major source of this interleukin in end-organs like the brain and kidney. In this scenario, the potent pro-inflammatory properties of the IL-17 as well as its direct action on the central nervous system suggest it as an activator of neurogenic mechanisms that contribute to SSH.

#### 3.3.2 IL-1β

IL-1β is a potent pro-inflammatory cytokine identified in 1984 (Auron et al., 1984) and primarily produced by activated macrophages. It mediates inflammatory responses, fever, and immune system activation. IL-1β is synthesized as an inactive precursor and is activated by caspase 1-mediated proteolytic cleavage, after what it can act on IL-1 receptors (Boraschi, 2022). Experimental findings have implicated IL-1β signaling with neurogenic mechanisms of the hypertension. Intracerebroventricular and PVN microinjections of IL-1β produce an increase in BP in normotensive Sprague-Dawley rats (Shi et al., 2011), indicating that pathways activated by IL-1β can play a role in hypertension. This signaling seems to include activation of perivascular macrophages in the brain tissue and increased expression/activation of type 2 cyclooxygenase (COX-2) thus leading to increased production of prostaglandin E_2_ (PGE_2_) (Yu et al., 2010). Heightened secretion of PGE_2_ by perivascular macrophages is believed to act in the PVN to increase the sympathetic drive to cardiovascular organs like the heart and vasculature (Yu et al., 2010). In a study carried out by Qi and cols., high salt diet (8% NaCl) increased BP, the biomarkers of inflammation within the PVN (including IL-1β expression) and norepinephrine plasma levels of DSS rats. Further, the bilateral microinjection of gevokizumab, an IL-1β inhibitor, in the PVN decreased the BP, the norepinephrine plasma levels, attenuated the levels of oxidative stress and restored the balance of cytokines within the PVN of DSS rats under high-salt diet (Qi et al., 2016). Corroborating these findings, both knockout of the IL-1 receptors (IL-1R) and pharmacological antagonism of IL-1R with anakinra (*i.p.*) reduced neurogenic pressor activity in deoxycorticosterone acetate (DOCA)-salt treated mice (Baumann et al., 2024). These findings indicate that central actions of IL-1β may be activating neuronal pathways controlling sympathetic drive to the cardiovascular system, thereby contributing to the neurogenic component of SSH.

#### 3.3.3 TNF-α

In addition to these findings, high salt intake has been shown to drive central action/production of tumor necrosis factor alpha (TNF-α) in animals with SSBP, particularly in the PVN. A study by Gao and cols. (2022) showed that TNF-α injection into the PVN trigged a dose and time-dependent increase in mRNA expression of PIM including IL-1β and IL-6, chemokines (C–C Motif Chemokine Ligand 5 (CCL5) and C–C Motif Chemokine Ligand 12 (CCL12)) as well as inducible nitric oxide synthase (iNOS), and NF-kB in cultured brain neurons from neonatal SD rats (Gao et al., 2022). The authors also assessed and compared mRNA expression of these genes at a basal level as well as in response to TNF-α challenge between SD rats and Dahl salt-sensitive rats. They found that cultured neurons presented higher baseline levels and greater response to TNF-α challenge in mRNA expression of CCL5, iNOS and IL-1β (Gao et al., 2022). The central administration of TNF-α caused higher mRNA expression response for CCL12 in the PVN of Dahl-S rats compared to SD rats (Gao et al., 2022), strongly suggesting high sensitivity of specific downstream TNF-α-activated PIM signaling pathways in the PVN of Dahl-salt rats. In addition, TNF-α mRNA expression in the PVN has been shown to increase in Dahl-salt sensitive rats under high salt diet (4% NaCl) and chronic intracerebroventricular infusions of oligodeoxynucleotide (ODN), an inhibitor of the inhibitory G protein family alpha subunit (Gαi_2_), indicating that G*α*i_2_ proteins mediate endogenous production of TNF-α in the PVN (Moreira et al., 2019), what may contribute to the development of SSH. The pieced evidence indicates that TNF-α signaling in the PVN of different hypertension models, including SSH models, is contributing to increased sympathetic drive (Moreira et al., 2019; Winklewski et al., 2015; Xiao and Harrison, 2020; Yu et al., 2019; Zera et al., 2009) further supporting the link between PIM signaling and neurogenic hypertension.

#### 3.3.4 IL-6

Heightened IL-6 levels are reported as one of the most consistent PIM changes associated with human hypertension (Afsar et al., 2018; Sesso et al., 2007). For instance, hypertensive patients have higher baseline IL-6 levels when compared to normotensives regardless of the salt intake (Chamarthi et al., 2011). Officially named in 1988 (Sehgal et al., 1989), IL-6 is an interleukin encoded by a pleiotropic gene and is involved with immune regulation, B cell maturation, T cell differentiation (especially T_H_17), inflammation, hematopoiesis, BBB permeability, and acute phase response (Grebenciucova and VanHaerents, 2023). IL-6 is produced by a wide array of cell types and is rapidly synthesized in response to infections, tissue injuries, and other inflammatory stimuli (Grebenciucova and VanHaerents, 2023). Interesting, IL-6 can produce effects reminiscent of a hormone, which include the normal homeostatic control of vascular function, lipid metabolism, insulin resistance, iron transport, mitochondrial activities. IL-6 acts as a neurotrophic factor, supporting differentiation, maturation and survival of neuronal subtypes like dopaminergic and cholinergic neurons (Bader and Meyer, 2022). In addition, IL-6 can affect neuronal excitability by regulating the expression and function of ion channels such as voltage-gated sodium and calcium channels, receptor/ligand-gated ion channels, and synaptic function and plasticity by influencing neurotransmitter release and synaptic strength (Grebenciucova and VanHaerents, 2023; Gruol, 2015). It is noteworthy that neuronal activity itself can stimulate the production of IL-6 in neurons (Sallmann et al., 2000). Further, IL-6 also acts as a mediator in neuroimmune interactions between neurons and glial cells, allowing communication during inflammatory states or infection (Grebenciucova and VanHaerents, 2023).

High salt intake is a stimulator of IL-6 production in PVN of Dahl salt-sensitive rats, and its increase has been correlated with hypertension development in this model (Du et al., 2020). In Sprague-Dawley rats under high salt intake, the upstream mechanism that stimulates not only IL-6, but IL-1β and TNF-α as well, in the PVN involves the suppression of the activation of the alpha subunit of the inhibitory G protein family (Gαi_2_). Since PVN is an important forebrain region involved with sympathetic drive to the cardiovascular system, the authors proposed that increased inflammatory state in the PVN, indexed by the increased expression of pro-inflammatory cytokines, is associated with increased sympathetic drive thus contributing to the hypertension in Dahl salt-sensitive rats. Two different studies showed that knockout mice for IL-6 were protected from the Ang II-induced hypertension (Huang et al., 2006; Hat et al., 2024) and one study showed that IL-6 knockout mice were protected from Ang II plus salt induced hypertension as well (Hat et al., 2024). Although some advances have been made for the understanding of the IL-6 role in the BP regulatory brain pathways, the underlying mechanisms remain largely unknown and require further investigation.

From the current knowledge in the field, high salt intake has been shown to increase the expression of important PIM like TNF-α, IL-1β and IL-6 in the PVN of Dahl-salt sensitive rats but not of Sprague Dawley rats (Jiang et al., 2018). This finding may indicate that a) the genetic background of Dahl salt sensitive rats prompt higher PIM production in key regions of the brain controlling BP and b) Dahl salt sensitive rats are more susceptible to diet-sodium driven PIM production in the central nervous system. However, another study showed that direct injection of high-sodium solution into the lateral ventricle of Sprague Dawley rats stimulated increased expression of TNF-α, IL-1β and IL-6 in the PVN and the exposure of cultured hypothalamic neurons to high extracellular levels of NaCl also increased TNF-α and IL-6 production (Jiang et al., 2018). Interesting, Deng and cols. also showed that high salt treatment caused activation of astrocytes both *in vivo* and *in vitro* in Sprague Dawley rats (Deng et al., 2017). Together, the findings seem to lead to the conclusion that, indeed, Dahl salt sensitive rats may develop an inflammatory state in brain that is more intense than the one found in Sprague-Dawley rats. Additionally, TNF-α, IL-1β and IL-6 expressions by astrocytes were increased in cell culture assays and the authors suggest that JAK/STAT3 plays an important role in this process (Deng et al., 2017). Further studies confirmed those findings and suggested that the TNF-α, IL-1β and IL-6 increase in the PVN is associated with significant microglia activation and oxidative stress in Dahl salt sensitive rats (Yu et al., 2022).

Many aspects and details of such mechanisms remain unclear, mostly because some effects of the cytokines in key sites of the brain are not fully understood. Taking together, the pieced evidence indicates that high salt intake stimulates pro-inflammatory cytokines in cardioregulatory centers of the brain to produce a neurogenic-mediated increase in BP suggesting that brain LGI may be an important factor leading to hypertension. However, studies have not addressed the question whether brain cytokines increase before or after hypertension develop in experimental models and in hypertensive patients, what might establish (or not) the causal relationship between LGI and SSH.

### 3.4 Blood-brain barrier disruption, low-grade inflammation and salt-sensitive hypertension

The BBB is a highly selective semi-permeable barrier that separates the brain from the bloodstream. It is composed of endothelial cells, astrocyte end-feet, and pericytes embedded in the capillary basement membrane (Abbott et al., 2010). The BBB regulates the transfer of solutes and chemicals between the circulatory system and the CNS, protecting the brain from harmful substances and preserving the tight ionic composition of the extracellular fluid while allowing essential nutrients to pass through (Abbott et al., 2010). Disruptions in the BBB have been linked to the development of autonomic and cognitive impairments (Katsi et al., 2020), which could contribute to hypertension. On the other hand, hypertension itself might also contribute to BBB disruption and to cognitive impairments development (Katsi et al., 2020; Faraco, 2024; Pelisch et al., 2011), which raises some questions about cause-consequence relationship between BBB disruption and hypertension development.

High salt intake has been shown to disrupt the BBB through different mechanisms which involve changes in the tight junction proteins, inflammatory responses, activation of sodium-sensitive signaling pathways in brain microvascular endothelial cells, and the renin-angiotensin system. In a model of permanent cerebral ischemia, mice under high salt intake displayed significant enhanced ischemic brain damage which was associated with enhanced BBB disruption, increased leukocytes infiltration and loss of tight junction (TJ) proteins (Zhang T. et al., 2015). The authors also found that high sodium concentrations downregulated tight-junction proteins expression by endothelial cells through a p38/MAPK/SGK1 pathway in immortalized murine brain microvascular endothelial cell line bEnd.3 (Zhang T. et al., 2015). These findings suggest that exposure of brain endothelial cells to high sodium chloride concentrations increase BBB permeability through such mechanisms. Interestingly, high salt intake has been shown to specifically increase sodium concentration in the CSF of the HS12W model (Gomes et al., 2017), Dahl salt sensitive rats (Nakamura and Cowley, 1989; Huang et al., 2004), and one-kidney, one-wrap renal hypertension model (Haywood et al., 1984; Leenen et al., 2002), without parallel changes in plasma sodium concentration. That would indicate that high sodium concentration in the brain can be achieved *in vivo*. Whether that level of sodium concentration is sufficient to drive the p38/MAPK/SGK1 pathway activation is still to be determined. In addition, Zhang and cols. (2015) also reported a positive correlation between urinary sodium levels and ischemic lesion size in stroke patients (Zhang T. et al., 2015), suggesting that BBB disruption by high-sodium intake may occur in humans as well.

The disruption of the BBB also involves a complex interplay between peripheral and central-generated PIM. One of the key players in this process is the high salt intake-related cytokine IL-17, which has been demonstrated to be involved with BBB disruption in an experimental autoimmune encephalomyelitis (EAE) model induced in C57BL/6 mice. In this model, IL‐17A induced the production of reactive oxygen species (ROS), which activated the endothelial contractile machinery. This activation was accompanied by a downregulation of the tight junction molecule occludin (Huppert et al., 2010), an important tight-junction protein in endothelial cells. Blocking either ROS formation or myosin light chain phosphorylation or sequestering IL-17 with IL‐17A‐neutralizing antibodies prevented IL‐17A‐induced BBB disruption (Huppert et al., 2010). A study by Kleinewietfeld also reported that high infiltration of T_H_17 cells into the CNS is present in a mouse model of experimental autoimmune encephalomyelitis (EAE) under high salt diet with co-involvement of the pro-inflammatory cytokines GM-CSF, TNF-α and IL-2 (Kleinewietfeld et al., 2013). In addition, cytokines like IL-1β, IL-6, IL-9, IL-17, INF-γ, TNF-α and CCL2 can impact tight-junction production and allocation, damage endothelial cells integrity, activate astrocytes and microglial cells and induce the penetration of peripheral immune cells into the CNS (Huang et al., 2021) through the disrupted BBB.

In addition to the role of immune-mediated mechanisms, disruption of the BBB has also been associated with high BP by itself (González-Marrero et al., 2012; Mueller and Heistad, 1980) and in some cases like the Ang II-induced high BP, BBB disruptions are reported to precede hypertension development in experimental models (Capone et al., 2011). Conversely, the majority of studies demonstrate disruption of the BBB after hypertension has been established, making it challenging to establish a causal relationship between BBB disruption, inflammation, and hypertension based on the current available knowledge. For instance, it has been demonstrated that capillary permeability of brain regions classically associated with sympathetic drive and BP control like PVN, NTS and RVLM are increased in hypertensive SHR but normal in pre-hypertensive SHR (Buttler et al., 2017). The injury process in vascular tissue stimulates the infiltration of inflammatory cells into the brain, especially in the perivascular tissue (Yu et al., 2010), reinforcing the LGI process. Once in the brain tissue, those cells are responsible for secreting other PIM such as IL-6, IL-1β and TNF-α locally (Hashmat et al., 2016; Winklewski et al., 2015) thus affecting local neurotransmission and neuronal activity of cardioregulatory centers in the brain. Consequently, it is plausible to speculate that salt-driven lesions in the BBB at critical sites for BP regulation can trigger the production of PIM within the brain in a positive feedback loop, thereby further activating neurogenic mechanisms associated with the development of SSH.

#### 3.4.1 Key points

1. CNS is important for BP regulation in both normotension and SSH, with neurogenic mechanisms contributing to elevating BP.
2. Cardiovascular reflexes like baroreflex and cardiopulmonary reflex are impaired or altered in SSH, with Dahl salt-sensitive rats showing reduced reflex sensitivity even under low salt intake.
3. The hypothalamic paraventricular nucleus (PVN) plays a key role in SSH and the immune signaling within the PVN, including microglial activation and proinflammatory cytokine production, are shown to largely contribute to high BP.
4. Though less studied, immune signaling in brainstem areas like RVLM and NTS also contribute to hypertension, although its role in SSH is still to be further explored.
5. High salt intake can disrupt the BBB and allow immune cells to infiltrate CNS. Higher immune cell infiltration in the CNS is associated with high BP, especially in the SSH.

## 4 The gut microbiome and the gut-brain axis in salt-sensitive hypertension

### 4.1 Gut microbiome

Over the past decade, a growing number of studies have allocated substantial resources to comprehending the impact of alterations in the gut microbiome on BP regulation (Palmu et al., 2021; Calderon-Perez et al., 2020; Canale et al., 2021; Cardoso, 2024; Marques et al., 2018; Pluznick, 2016). A portion of these studies have specifically focused on the effects of excessive salt consumption on the changes in gut microbiome and its subsequent influence on BP regulatory mechanisms (Palmu et al., 2021; Canale et al., 2021; Abais-Battad et al., 2021; Chrysant, 2024; Elijovich et al., 2020; Mu et al., 2024). A prevailing consensus among various authors is that excessive salt consumption adversely affects the host’s health by diminishing the α and β diversity of the gut microbiome and leverage the balance toward the predominance of bacterial species that promote an inflammatory state and the production of detrimental metabolites (Wilck et al., 2017; Calderon-Perez et al., 2020; Elijovich et al., 2020; Mu et al., 2024; Mell et al., 2015; Xu et al., 2022; Yin et al., 2024). The imbalance between health-promoting and disease-causing gut bacteria, known as dysbiosis, is theorized to have a substantial impact on BP regulation through the gut-brain axis signaling system thus contributing to SSH in experimental models and humans (Elijovich et al., 2020; Nagase et al., 2020).

The gut microbiome produces several metabolites with bioactive properties that influence brain chemistry, stress response, cognitive and autonomic functions of the host. In this context, gut microbiome dysbiosis has been associated with pathophysiological mechanisms that may cause cardiovascular diseases. Indeed, high salt intake results in dysbiosis in the gut microbiome. This has been shown to cause the host’s differentiation of naïve T cells into pro-inflammatory T_H_17 cells, shifting the immune system towards a pro-inflammatory state, and promote the production of pro-hypertensive short-chain fatty acids (SCFA) by specific groups of gut bacteria (Wilck et al., 2017; Wang et al., 2022; Abais-Battad et al., 2021; Elijovich et al., 2020; Xu et al., 2022; Yin et al., 2024; Chakraborty et al., 2018; Chen et al., 2020). Such events in the gut affect the inflammatory signaling mechanisms within different organs controlling BP, which include the brain and the kidneys. These mechanisms have been actively discussed within the concept of signaling axis that involve mostly the gut, brain and kidneys. The gut-brain axis has received considerable attention recently due to its ability to influencing BP control, especially in SSH. By the current definition, the gut-brain axis refers to the bidirectional communication network between the gastrointestinal tract (GI tract) and the CNS. This complex system involves multiple key pathways, including neural, endocrine, immune, and humoral links which influence each other’s functions and BP levels (Carabotti et al., 2015; Mell et al., 2015). Evidence has shown that the gut-brain axis affects neural mechanisms controlling BP. Although much has been investigated on the correlation between hypertension and gut microbiome dysbiosis, the pathophysiological mechanism through which it occurs remains mostly unknown. Whether high salt intake can cause neurogenic hypertension through increased sympathetic outflow due to gut microbiome dysbiosis is yet to be further investigated. However, evidence shows that IL-17, a product of T_H_17 cells that proliferate under gut microbiome dysbiosis (Wilck et al., 2017), can act in the PVN to increase sympathetic outflow (Cao et al., 2021), and corroborates the theory that dysbiosis may be causally related to neurogenic SSH.

The study by Wilck et al. (2017) has shown that a 4% NaCl diet for 14 days caused a reduction in the *L. murinus* population of mice intestinal microbiota (Wilck et al., 2017). Such change was accompanied by increased BP and increased population of CD4 T_H_17 pro-inflammatory cells in the intestinal mucosa (Wilck et al., 2017). When high-salt-fed mice were switched back to a regular salt diet, the intestinal population of *L. murinus* as well as BP returned to normal, strongly suggesting that the impact of high salt intake on *Lactobacillus murinus* population: a) is correlated with hypertension in these animals and b) are reversible (Wilck et al., 2017). A study conducted by Bier and colleagues (2018) also showed that excessive salt consumption elevated the relative abundance of the taxa belonging to the *Erwinia* genus, *Christensenellaceae*, and *Corynebacteriaceae* families in Dahl salt-sensitive rats (Bier et al., 2018) suggesting the existence of “halophilic” bacteria in the gut microbiome. Conversely, it also reduced the abundance of the *Anaerostipes* genus in this model (Bier et al., 2018). These findings suggest that, beyond the *Lactobacillus* genus, the abundancy of other genera and even entire families of the gut microbiome can be impacted by high salt intake. In addition, previous (Linz et al., 2012; Pacha, 1998) and recent (de Assis Braga et al., 2025) studies also showed that high salt intake can, indeed, increase the content of sodium in the feces *in vivo* further supporting the hypothesis that, despite the vast majority of sodium intake is absorbed by the small intestine, a significant amount of sodium reaches the large intestine and function as selective pressure mechanism of the environment on gut microbiome. Such changes appear to be correlated with a shift in the relative abundance of specific families and genera that favor specific pro-hypertensive bacterial groups over rather than a general and unspecific impact on the gut microbiome of SSH models (de Assis Braga et al., 2025).

Corroborating experimental data, a recent study has described dysbiosis of the intestinal microbiota in hypertensive patients. This dysbiosis was characterized by reduced biodiversity and distinct bacterial signatures compared to the normotensive counterpart (Silveira-Nunes et al., 2020). Along with a reduction in Bacteroidetes members, hypertensive individuals displayed increased proportions of *Lactobacillus* and *Akkermansia* and decreased relative abundances *Roseburia* and *Faecalibacterium* within the *Lachnospiraceae* and *Ruminococcaceae* families (Silveira-Nunes et al., 2020). The increased proportion of *Lactobacillus* genera in hypertensive patients may indicate that, different of experimental findings in mice, the reduction in abundance of the genus *Lactobacillus* is not essential for the hypertension in humans. This study also reported a pro-inflammatory immune profile in hypertensive individuals with an increase in TNF/IFN-γ ratio, and in TNF and IL-6 production in peripheral blood when compared to normotensive subjects (Silveira-Nunes et al., 2020). Despite lending support for experimental data, this study did not address important questions like whether the changes in gut microbiota are, indeed, a cause for the hypertension in humans or if dietary salt consumption was correlated with the reported changes in microbiota. This question was addressed in a paper by Nagase et al. (2020) which concluded that consumption of low-salt diet was ineffective in regulating hypertension in individuals with a specific gut microbiome composition (Nagase et al., 2020). Therefore, the authors defend the idea that restoration of the gut microbiome should be considered a new better approach for controlling BP and preventing hypertension in humans (Nagase et al., 2020).

### 4.2 Short-chain fatty acids

Short-chain fatty acids (SCFA) have been recognized as important molecules from gut microbiome metabolism that play a significant role in the gut-brain axis signaling system. By definition, SCFA are a group of saturated aliphatic organic acids with a chain length of one to six carbon atoms. The most common SCFA produced by gut microbiome are acetate (C2), propionate (C3), and butyrate (C4), which are produced primarily through the anaerobic fermentation of indigestible polysaccharides, such as dietary fiber and resistant starch (Silva et al., 2020). The ratio of acetate:propionate:butyrate in the colon has been estimated in 3:1:1, meaning that acetate is the most abundant SCFA produced (He et al., 2020; Liu et al., 2021). The SCFA, produced by luminal large intestine bacteria, can enter the body through monocarboxylate transporters (Coady et al., 2004) or through simple diffusion (Charney et al., 1998). The SCFA plasma concentration in the host is, however, considerably smaller than colonic concentration. For instance, acetate has a typical plasma concentration about 1,000 to 2,000 times smaller (50–100 μmol/L) than colonic concentration (100 mmol/L), albeit it is still substantial (Xu et al., 2022). Acetate (produced by most of the *Enterococcus* species) and propionate (produced by Bacteroidetes, *Acidaminococcus* and *Salmonella*) are easily absorbed and transported to the liver. In the liver, propionate is known to promote the intrahepatic gluconeogenesis (Xu et al., 2022). In addition, bacteria from the genera *Clostridium*, *Eubacterium* and *Roseburia* are known as the major sources of butyrate (Liu et al., 2021; Wong et al., 2006; Nicholson et al., 2012; Louis and Flint, 2017; Sasaki et al., 2020).

The role of SCFA and mechanisms in the SSH is yet to be further explored. However, different studies have pointed out some beneficial functions for SCFA in the host´s health. For instance, Wu and cols. (2021) showed that oral administration of the SCFA sodium butyrate attenuated the hypertension and renal damage in DOCA/salt rats by a mechanism that involves inhibition of the MR/SGK1 pathway (Wu et al., 2021). Additionally, dietary sodium reduction has been shown to increase circulating SCFA which are associated with decreased BP and improved arterial compliance (Chen et al., 2020). On the other hand, high salt intake has been shown to reduce the concentrations of SCFA such as acetate, propionate, and butyrate in fecal samples of mice fed 8% salt diet, what was associated with cognitive dysfunction and gut dysbiosis (Hu et al., 2020). However, Dahl salt sensitive rats displayed increased levels of fecal acetate, propionic acid and isobutyrate, but not butyrate, when fed a 4% salt diet (Bier et al., 2018), contrasting with the high-salt intake findings in mice. A study by Bruning and cols. (2020) also raised some issues regarding the benefits of SCFA on BP regulation. The authors reported that microinjection of the acetate into the PVN of anesthetized rats produced an increase in splanchnic sympathetic nerve activity and BP (Bruning et al., 2020). Also, the pre-injection of kynurenic acid, an ionotropic EAA receptor antagonist, attenuated the sympathoexcitation and BP increase produced by acetate (Bruning et al., 2020), strongly suggesting that the acetate-induced excitation in the PVN could involve EAA neurotransmission.

SCFA produce their biological effects in the host by activating specific G protein-coupled receptors expressed throughout the body such as GPR41 and Olfr78, which are expressed in blood vessels and other tissues. Evidence from whole body knockout mice showed that the lack of GPR41 receptors led to hypertension in these animals while Olfr78 knockout mice are normotensive (Xu et al., 2022). Also, the SCFA GPR109A receptor in the rostral ventrolateral medulla (RVLM) plays a role in central BP control, where activation by nicotinic acid leads to l-glutamate release, subsequently increasing sympathetic activity and BP (Rezq and Abdel-Rahman, 2016). In addition, the EC_50_ values for acetate, propionate and butyrate are largely different for the GRP41, GRP43 and Olfr78 receptors (Xu et al., 2022; Lu et al., 2022; Pluznick, 2014). For instance, acetate has the lower affinity while propionate and butyrate have higher affinity for SCFA receptors (Table 2). This pharmacological aspect of the SCFA may also, at least in part, contribute to different and, sometimes, conflicting effects of SCFA on BP. Together, these findings picture a complex interaction between SCFA production and host’s response to it, especially concerning SSH. However, studies in humans have shown that dietary sodium reduction increases circulating levels of 2-methylbutyrate, butyrate, hexanoate, isobutyrate, and valerate SCFA and that such an increase in circulating SCFA was associated with BP reduction and improvement of arterial compliance (Chen et al., 2020; Ríos-Covian et al., 2016). Further studies are needed to establish whether SCFA effects on autonomic control of BP are specific to each individual molecule, the combination of them or if it is dependent on the major action site in the brain. A summary of the major findings is shown in Table 2.

**TABLE 2:** Major short chain fatty acids produced by the gut microbiome and the effects on blood pressure regulation.

Short chain fatty acid (SCFA)	Molecular structure	Bacterial source	Effect on blood pressure	EC_50_ values (μM) for the major SCFA receptors			Ref.
				GPR41	GPR43	Olfr78	
Acetate	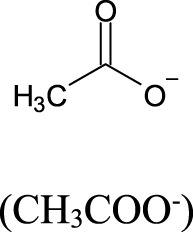	Produced primarily by *Bifidobacterum* spp., *Akkermansia* spp., and some Bacteroidetes species by fermentation of dietary fibers	Generally, associates with lowering BP through GPR41-mediated vasodilation; can also increase BP through Olfr78-mediated renin release, indicating a dual/buffering effect	393–1,072	35–431	2.01–2.35	Xu et al., 2022; Pluznick (2014), Fusco et al. (2023), Li et al. (2019), Liu et al. (2023)
Propionate	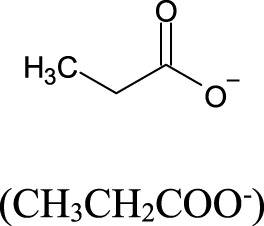	Produced mainly by Bacteroidetes, Veillonella and some Firmicutes species by fermentation of carbohydrates	Lowers BP through GPR41 activation and vasodilation; can also increase BP through Olfr78 receptor activation and renin release, but the overall effect is hypotensive in most studies	14–290	14–290	0.63–0.92	Xu et al., 2022; Pluznick (2014), Fusco et al. (2023), Li et al. (2019), Liu et al. (2023)
Butyrate	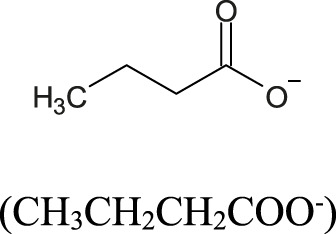	Produced predominantly by Firmicutes (e.g., *Faecalibacterium prausnitzii*, *Roseburia* spp., *Eubacterium* spp., *Clostridium clusters* IV and XIVa)	Lowers BP by activation of GPR41 and influence on the autonomic nervous system (notably via vagal afferents); also promotes regulatory T cell differentiation, contributing to anti-inflammatory effects	33–158	28;– 371	Not reported	Xu et al., 2022; Pluznick (2014), Fusco et al. (2023), Li et al. (2019), Liu et al. (2023), Furusawa et al. (2013)

EC_50_, effective concentration for 50% of maximal effect; higher values of EC_50_ indicate lower affinity of the SCFA, for the receptor binding site; µM, micromolar; GPR41, SCFA G protein-coupled receptor 41; GPR43, SCFA G protein-coupled receptor 43; Olfr78, olfactory G protein-coupled receptor 78 (senses low oxygen levels in the carotid body and responds to short-chain fatty acids produced by gut microbiota in the colon).

#### 4.2.1 Key points

1. High salt intake can influence gut microbiome composition.
2. Composition of the gut microbiome can affect BP regulation by mechanisms involving inflammatory signaling and detrimental metabolite production.
3. The gut-brain axis, a bidirectional signaling network between the gut and the CNS, plays a role in BP control.
4. Dysbiosis in the gut microbiome can lead to increased sympathetic outflow and neurogenic hypertension.
5. Experimental data shows that high salt intake can cause dysbiosis shifting the Firmicutes/Bacteroidetes ration which is correlated with high BP and pro-inflammatory state.
6. In humans, dysbiosis in hypertensive patients is characterized by reduced biodiversity and distinct microbiome composition signature compared to normotensive individuals.
7. Short-chain fatty acids (SCFA), produced by gut microbiome, play a role in BP regulation. While some studies show beneficial effect of SCFA on BP, others indicate potential adverse effects, highlighting the complexity of their role in hypertension.

## 5 Kidney, salt-sensitive hypertension and inflammation

Sodium balance is tightly regulated by kidneys under a wide range of sodium intake (Bagordo et al., 2024). Although recent evidence indicates that both human and rodents display nonosmolar sodium retention in skin and skeletal muscle under high salt intake (Thowsen et al., 2022; Titze, 2014), the kidneys still have a dominant role in the sodium handling by the body. The traditional view that high salt intake can lead to hypertension because of increased water intake followed by plasma volume expansion, increased cardiac output and autoregulatory vasoconstriction has been challenged by current evidence showing that plasma volume does not increase under high salt intake that mimics the human setting in a rodent model of SSH (Gomes et al., 2017; de Souza et al., 2021). The results of these studies suggest that hypertension is initially driven by neurogenic mechanisms with renal histological and functional disfunctions after the establishment of the hypertension (de Souza et al., 2021).

The accumulation of sodium in tissues has been demonstrated to contribute to systemic LGI (Sahinoz et al., 2021) which can impact renal functions through a variety of mechanisms (Frame and Wainford, 2017). For instance, the infiltration of immune cells in the kidneys has been shown to play an important role in the renal mechanisms that contribute to SSH in experimental models (Mattson, 2014). The genetic deletion of the recombination-activating gen 1 (Rag1) in Dahl-salt sensitive rats resulted in a significant reduction in T and B cells in the blood and spleen under normal salt diet (Mattson et al., 2013). Also, the exposure of these animals to high salt diet produced a blunted infiltration of T cells into the kidney that is typically found in wild type Dahl salt sensitive rats following high salt intake (Mattson et al., 2013). The reduction of T cells infiltration into the kidneys of Rag1-null Dahl salt sensitive rats was accompanied by a significant reduction in the SSH of these animals (Mattson et al., 2013). Furthermore, the infiltration of immune cells into the kidneys of Wistar rats under high salt diet following l-NAME administration was described as responsible for the impaired pressure natriuresis that contributes to SSH (Franco et al., 2013). The progression of renal injury in obese Dahl salt sensitive leptin mutant rats was associated with increased expression of the macrophage inflammatory protein 3-α (MIP3α) and, consequently, increased immune cell recruitment and infiltration in the renal tissue, thus producing renal low-grade inflammation (Ekperikpe et al., 2023). Interestingly, high salt, but not high BP, induces immune cell activation, renal infiltration, high expression of Na^+^/K^+^-ATPase and SSH in ovariectomized rats (Vlachovsky et al., 2024) showing that high salt intake has a more prominent effect on immune kidney system than high BP alone.

As immune cells infiltrate the kidneys, PIM drive important changes in the expression and activity of sodium channels in the nephron affecting sodium handling by kidney. This was be demonstrated in an AngII-driven hypertension mouse model in which i) RAS-mediated hypertension was accompanied by elevated levels of the macrophage cytokine IL-1 in the kidney (Crowley et al., 2010) and ii) the IL-1R deficiency or blockade limits BP elevation in this model by mitigating sodium reabsorption via the NKCC2 co-transporter in the nephron through a mechanism dependent on nitric oxide production by intra-renal macrophages (Zhang et al., 2016). The authors also showed that sodium balance in the IL-1R knockout mice became negative under Ang-II infusion while sodium balance in wild type mice increased, and that pre-treatment with furosemide abrogated the difference. Given that sodium balance is a reliable indicator of natriuresis relative to sodium intake, the findings indicate that IL-1R may contribute to the Ang-II-induced sodium retention, as proposed by the authors (Crowley et al., 2010). However, plasma sodium concentrations were not addressed in this work, leaving questions on whether IL-1 cytokine is actually influencing extracellular fluid concentration of sodium and/or affecting sodium retention in the skin and muscle (Titze, 2014), extra or intracellularly (Thowsen et al., 2022) following high sodium intake. Further evidence indicates that IL-1β plays a direct role in SSH and this is supported by findings that inflammasome components NLRP3, ASC, and caspase-1 were not only present in distal tubules and collecting ducts of Dahl-salt sensitive rats but also increased under high salt intake (Zhu et al., 2016). Additionally, the infusion of the caspase-1 inhibitor Ac-YVAD-cmk as well as the transplantation of mesenchymal stem cells into the renal medulla of Dahl-salt sensitive rats reduced the NLRP3 inflammasome activation and the salt-induced hypertension (Zhu et al., 2016), linking the inflammasome-derived mature IL-1β signaling system in the kidney to the SSH. Further supporting the role of IL-1β on renal sodium handling, diabetic db/db mice harboring an IL-1 receptor type 1 knockout bone marrow (db/db^IL1RKO^) phenotype displayed lower expression and activity of epithelial sodium channel (ENaC) expression and activity compared with db/db transplanted with a wild-type bone marrow (Veiras et al., 2022). The db/db mice features high levels of kidney cortical IL-1β expression and, alongside with diabetes, have progressive increase in BP levels when exposed to high salt diet which involves an impaired downregulation of ENaC in the kidney epithelial cells in response to high sodium (Veiras et al., 2021). When exposed to high salt intake, the db/db^IL1RKO^ displayed a salt resistant BP as well as lower renal inflammation with reduced IL-1β-induced production of IL-6 by macrophages in the kidneys (Veiras et al., 2022). These findings indicate that kidney cortical IL-1β overproduction increases renal ENaC expression and activity through a mechanism that involves macrophages polarization toward a proinflammatory phenotype and renal IL-6 accumulation, with concurrent salt sensitivity of the BP (Veiras et al., 2022).

Chronic infusion of Ang II is well known for its ability to increase BP levels and significantly decrease natriuresis when experimental animals are challenged with saline solutions. Also, Ang II regulates the synthesis of pro-inflammatory cytokines and chemokines in the kidneys of female Wistar rats (Ruiz-Ortega et al., 2002) and can increase abundance of the phosphorylated forms of the Na-K-2Cl cotransporter (NKCC), Na-Cl cotransporter, Ste20/SPS-1-related proline-alanine-rich kinase in the distal tubule as well as ENaC expression in the collecting duct in mice (Kamat et al., 2015). Parallel to its direct effects on renal expression of sodium handling ion channels, Ang II, DOCA-salt and norepinephrine cause T cells and monocytes/macrophages to accumulate in the kidney and increase the local production of pro-inflammatory cytokines like INF-γ and IL-17 (Itani and Harrison, 2015). In this context, knockout mice for INF-γ (IFN-γ(−/−)) or IL-17A (IL-17A (−/−)) displayed a blunted response to Ang II-induced hypertension (Kamat et al., 2015) indicating that Ang II-driven hypertensin also involves downstream pathways activated by INF-γ and IL-17 cytokines. However, INF-γ(−/−) and IL-17 (−/−) did not affected the Ang II-induced increase in abundance of the phosphorylated forms of the Na-K-2Cl cotransporter, Na-Cl cotransporter, Ste20/SPS-1-related proline-alanine-rich kinase in the distal tubule but significantly decreased the abundance of Na/H-exchanger isoform 3, sodium phosphate transporter isoform 2 (NaPi2) and the motor myosin VI in the proximal tubule (Kamat et al., 2015). These findings suggest that both INF-γ and IL-17 can interfere with the renal sodium handling by altering the expression of sodium transporters within the initial portion of the nephron. Given the total amount of filtered sodium reabsorbed in the portion of the nephron, even small changes in ENaC expression could have a significant impact on sodium retention and its impact on BP.

The exposure of ENaC in M-1 cortical collecting duct cells to IL-6 increased the protein expression of α-ENaC, β-ENaC, γ-ENaC and prostasin as well as the amiloride-sensitive sodium current in a study by Li and cols. (2010) (Li et al., 2010). The SSBP is also strongly correlated with the T-lymphocytes levels of mRNA for IL-6 isolated from kidneys when compared to circulating T-lymphocytes in Dahl salt sensitive rats (Rudemiller et al., 2014). By treating these animals with an IL-6 neutralizing antibody, Hashmat and cols. (2016) showed that a decreased renal cortical level of IL-6 was correlated with reduced glomerular and tubular damaged as well as with BP levels in Dahl salt sensitive rats under high salt intake (Hashmat et al., 2016) suggesting the IL-6 may exacerbate may be involved in the salt sensitivity of Dahl salt sensitive rats.

The role of TNF-α in the renal changes that occur in response to high salt intake challenge are particularly interesting, especially because of the poor understanding of the exact mechanisms underlying the role of TNF-α in the SSH. While TNF-α antagonism has attenuates the hypertensive responses in many hypertensive animal models, contrasting findings demonstrate that the direct systemic administration of TNF-α usually induces hypotensive as well as natriuretic responses (Mehaffey and Majid, 2017) indicating that the solo increase in the circulating TNF-α levels may oppose the SSH. Such contrasting outcomes are speculated to result, at least in part, from differential activities of the two TNF-α cell surface receptors expressed in the kidney tissue. The TNFR1 is usually found in the proximal tubule, collecting duct, vascular endothelium and vascular smooth muscle while TNFR2 are usually found in the proximal tubule, collecting duct, vascular endothelium but not in the vascular smooth muscle (Mehaffey and Majid, 2017; Castillo et al., 2012). Studies conducted in TNFR1 knockout (TNFR1KO) and TNFR2 knockout (TNFR2KO) mice have elucidated the differential effects of both receptors on renal sodium handling and renal injury induced by TNF-α. The TNF-α infusion in mice triggers a surge in urinary volume output and fractional excretion of sodium, while the absence of TNFR1 attenuates this response (Castillo et al., 2012). Also, the TNF-α natriuretic activity is amiloride-sensitive suggesting that such effect may be mediated by ENaC activity (Majid, 2011; Shahid et al., 2008). Corroborating the role of TNFR1 role in renal sodium handling, recent findings showed that TNFR1 activity is downregulated in eNOSKO mice, which facilitates salt retention and contribute to SSH under high salt intake (Majid et al., 2024). On the other hand, mice lacking TNFR2 displayed less Ang II-induced fibrotic changes and macrophage extravasation indicating that TNF-α action on TNFR2 promotes renal tissue damage through proinflammatory pathway (Mehaffey and Majid, 2017; Singh et al., 2013). In summary, the actions of TNF-α in the kidney strongly depend on the receptor type it acts on and may oppose the sodium retention in mice under high-salt intake, probably counteracting its pro-hypertensive effects. That would strongly indicate that the role of TNF-α may depend on the production of other cytokines and chemokines in SSH.

The immune events that originate in the kidneys not only affect local renal functions but also influence immune events that occur in the brain. The renal and central nervous system signal each other through afferent (sensory) and efferent (motor) sympathetic autonomic nerves and recent findings suggest that signaling system has an important role in the SSH. Using the rat DOCA-salt model, Banek and cols. Showed that afferent renal nerve activity is augmented and that both, total and selective afferent renal nerves ablation, attenuated the DOCA-salt induced hypertension by about 50% (Banek et al., 2016). In addition, the authors also found that total, but not selective, afferent renal denervation attenuated the DOCA-salt induced renal inflammation assessed by CD3^+^, CD4^+^ and CD8^+^ cell counting and inflammatory markers (GRO/KC, MCP-1, IL-1β, IL-2, IL-6, IL-17a, and TNF-α) in the kidney (Banek et al., 2016). The findings suggest that while afferent renal nerves contribute to hypertension in the rat DOCA-salt model, the efferent renal nerves contribute to renal inflammation. The authors also proposed that renal inflammation may drive afferent renal activity to the central nervous system thus contribute to a positive feedback loop to the renal inflammation and to hypertension. Further studies on DOCA-salt model showed that the IL-1R activation is partially responsible for the increased afferents renal nerve activity and that IL-1β is increased in the kidneys and urine of DOCA-salt mice (Baumann et al., 2024). As in the DOCA-salt model of hypertension, both total renal denervation and afferent renal denervation attenuated the hypertension in the 2 kidneys, one clip (2K1C) model (Lauar et al., 2023). In addition to increased pro-inflammatory cytokines (TNF-α and IL-1β) in the clipped kidney and urine, the authors found increased expression of these cytokines in the hypothalamus of 2K1C rats. Curiously, both, total and afferent renal denervation decreased the levels of TNF-α, but only afferent renal denervation decreased the levels of IL-1β in the hypothalamus of 2K1C rats (Lauar et al., 2023), suggesting that renal inflammation can trigger an immune response in the central nervous system of this hypertension model. Together, the findings indicate a complex reinforcing interplay between the kidneys and the central nervous system to sustain SSH through immune-mediated mechanisms.

### 5.1 Key points

1. The kidneys play a dominant role in regulating sodium balance in the body, even under high salt intake. Recent evidence challenges the traditional view that high salt intake led to hypertension through increased plasma volume and suggests that hypertension due to high salt intake is initiated by neurogenic mechanisms.
2. Sodium accumulation in tissues contribute to systemic low-grade inflammation which may impact renal function. Genetic studies implicate increased immune cell infiltration in the kidney as a mechanism that contribute to SSH specially because hypertension is reduced when immune cell infiltration is blunted.
3. IL-1 influences sodium retention and hypertension since IL-1R deficiency or blockade limits BP elevation by reducing sodium reabsorption in the nephron.
4. Ang II regulates pro-inflammatory cytokines production in the kidneys leading to renal damage and altering sodium transporters expression.
5. Renal and central nervous systems communicate to each other through afferent and efferent sympathetic renal nerves contributing to SSH. Inflammatory signaling in the kidneys trigger immune responses in the brain that further contribute to sympathetic stimulation, creating a positive feedback loop that contributes to SSH.

## 6 Age and low-grade inflammation in the salt-sensitive hypertension

Aging is an inevitable ongoing process that is part of life and is also an important risk factor for cardiovascular disease (North and Sinclair, 2012). Despite its importance for cardiovascular diseases, the underlying mechanisms behind the processes that build up age-related impairments to the cardiovascular system are not fully understood. However, it is important to point out that the SSBP is a trait that becomes more pronounced as individuals age (Osanai et al., 1993) and, therefore, can be regarded as an additional risk factor for those carrying this trait. The association between age and SSBP has been documented and has been linked to vascular dysfunction and diminished kidney function. Factors as epigenetic modifications, diet, gut microbiome abundancy and diversity, intrinsic immune system setting, mitochondrial dysfunctions, increased levels of cortisol, immunosenescence and, regarding women, menopause are the prevailing elements that largely contribute to age-related cardiovascular disease (Demirci et al., 2025).

Recent experimental studies have shown that the aging process in Sprague-Dawley (SD) rats from 3 to 16 months is accompanied by increased BP, enhanced neurogenic pressor activity, BBB disruption, increased PIM (IL-6 and TNF-α) production, and microglia activation in the PVN of male Sprague-Dawley rats (Nist et al., 2024). These findings indicate that aging is associated with increased brain production of pro-inflammatory molecules in the PVN, a key brain region controlling sympathetic nerve activity and BP of male rats. The authors also found that treatment with losartan, an AT1R antagonist used as first-line treatment of hypertension, improved BP levels as well as reduced microglia activation and IL-6 and TNF-α production in the PVN of 16 months old male rats (Nist et al., 2024). Interestingly, females SD rats lack the increased BP and increased PIM production in the PVN during the same aging period indicating that female SD rats do not undergo the same cardiovascular issues that male SD rats due to aging. On the other hand, earlier studies showed that Dahl salt sensitive female rats, even under low-sodium diet, displayed an increase in BP over time, from the age of 3 months to the age of 12 months (Hinojosa-Laborde et al., 2004) indicating that the salt sensitive trait in these animals may overcome the protective effects of sexual hormones during the evaluated aging period. Ovariectomy accelerated the development of hypertension in Dahl salt rats, while the ovariectomy accompanied by estrogen replacement attenuated its development (Hinojosa-Laborde et al., 2004). These findings are aligned with results from studies showing an increase in salt sensitivity over the aging process is also reported in humans (Sander, 2002; Schulman et al., 2006) and further highlight the importance of the steroid hormones in the SSBP protection as salt-resistance women pre-menopause become salt-sensitive after menopause (Schulman et al., 2006).

A growing number of studies have showed that the decline in expression of the Klotho protein is linked to aging in humans and experimental animals (Abraham and Li, 2022). Klotho is a membrane/soluble protein expressed in the choroid plexus of the brain, a structure primarily linked to the CSF production, and the convoluted tubules in the kidney (Abraham and Li, 2022). The circulating levels of the soluble form of Klotho (α-Klotho) have been shown to directly correlate with glomerular filtration rate suggesting its diagnostic importance in kidney function decline, especially during aging (John et al., 2011). Interesting, the missense single nucleotide polymorphism in the Klotho gene, rs9536314, do humans is also associated with SSH (Citterio et al., 2020). In addition, experimental findings showed that high sodium intake led to SSH in mice Klotho heterozygous knockout mice as wells as in aged mice and that Klotho supplemented reversed SSH in these animals (Kawarazaki et al., 2020). Also, Klotho deficiency in heterozygous knockout mice is associated with renal damage and SSH through CCR2-mediated inflammation (Zhou et al., 2015), linking the Klotho deficiency with immune regulation of SSBP. Whether Klotho blood levels can function as a reliable marker of SSBP, especially in aged people, is yet to be determined.

The ongoing findings point toward the important role of immune signaling in key brain regions controlling autonomic regulation of BP in experimental models and may represent an important step for the understanding of its role in the human set of SSBP.

### 6.1 Key points

1. Aging is an important risk factor for cardiovascular diseases that encompass factors like epigenetic modifications, diet, gut microbiome changes, immune system setting, mitochondrial dysfunctions, hormones changes and other factors that contribute to age-related BP elevation over time.
2. Experimental data have shown that aging is associated with hypertension development along with increased neurogenic pressor activity, BBB disruption and low-grade neuroinflammation.
3. Interesting, female experimental animals did not display the same increased BP associated with PIM production as male during aging. However, the salt-sensitive trait found in Dahl-salt sensitive rats resulted in increased BP during aging suggesting this trait may overcome the protective effects of female sexual hormones.
4. The decline in Klotho protein expression is linked to aging and SSH. The mechanisms seem to involve renal function decline and inflammation.

## 7 Therapeutic implications and future directions

High salt intake is important risk factor for cardiovascular and kidney disease and yet reductions in salt intake have proved a challenging task to be achieved in the modern western society. Although the daily salt consumption has, indeed, reduced from the 18th century to current days (Ha, 2014), it still far from the recommendations by worldwide health authorities. Therefore, new strategies and approaches must be considered to mitigate the effects of the high salt intake, especially when considering the aging and longer lifespan our society conquer over the past century. The gut microbiota plays a crucial role in regulating BP and kidney function through its interaction with the immune and nervous systems. Therapeutic strategies such as dietary interventions, probiotics, prebiotics, symbiotic, and fecal microbiota transplantation have shown promise, under experimental and controlled conditions, in modulating gut microbiota to manage hypertension and chronic kidney disease (Al-Habsi et al., 2024; Baldi et al., 2021). These interventions aim to restore microbial balance, enhance gut barrier function, and reduce systemic inflammation, which are critical for lowering BP and protecting kidney and brain health. Targeting neural pathways that regulate immunity, and inflammation offers a novel approach to treating kidney diseases and hypertension. The use of anti-inflammatory agents that specifically target the CNS like minocycline or drugs with specific action on cytokine receptors like IL-1R and TNFR antagonists may be proven effective in counteract the pro-inflammatory effects of high salt intake and work alongside conventional anti-hypertensive therapies. SCFA, produced by gut microbiota, have anti-inflammatory effects and play a role in BP regulation. Enhancing SCFA production through dietary modifications or supplementation could be a therapeutic strategy to mitigate hypertension and its associated kidney damage.

However, further research is needed to elucidate the precise mechanisms by which the gut microbiota influences hypertension and kidney diseases. Understanding these pathways will aid in the development of targeted therapies that can more effectively modulate the neuron-immune-microbiome axis and may mitigate the detrimental effects of high salt intake on the cardiovascular and renal systems. As our understanding of the microbiome’s role in hypertension and kidney diseases grows, there is potential for developing personalized treatment strategies. These could involve tailoring dietary interventions and microbiota-targeted therapies based on individual microbiome profiles. Conducting well-designed clinical trials to test the efficacy of microbiota-targeted interventions in humans is crucial. These trials will help validate the therapeutic potential of probiotics, prebiotics, and other microbiome-related strategies in managing hypertension and salt-related kidney diseases. In addition, utilizing metagenomics, metabolomics, and other omics technologies can provide comprehensive insights into the microbiome’s role in disease pathogenesis. This integration will facilitate the identification of novel biomarkers and therapeutic targets.

## 8 Conclusion

**FIGURE 1:**
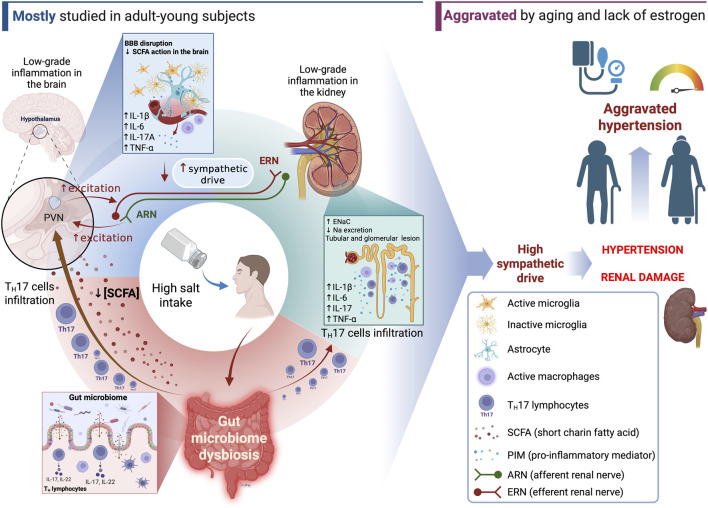
Summary of the major findings and working hypothesis for some of the mechanisms underlying the SSH. Gut microbiome dysbiosis can influence T_H_ cell differentiation and the chemical diversity/quantity of the short chain fatty acids produced by bacterial metabolisms. These changes can promote immune responses in the central nervous system (particularly in the paraventricular nucleus of the hypothalamus–PVN) that shift the autonomic control of the BP toward a more active sympathetic drive. In addition, high salt intake and gut dysbiosis can induce immune signaling and immune cell infiltration in the kidney, affecting sodium handling and renal function. Together, those changes can favor an increase in total peripheral resistance and sodium/volume retention over time, largely contributing the hypertension development. It is noteworthy that age and sex differences can influence the ways such changes take place and aggravate hypertension under these low-grade inflammation state, further leading the higher levels of BP. SCFA, short chain fatty acids; PIM, pro-inflammatory cytokines; ARN, afferent renal nerve; ERN, efferent renal nerve; IL-1β, interleukine 1β; IL-6, interleukine 6; IL-17A, interleukine 17A; TNF-α, tumor necrosis factor α.

In this review, we focused on key findings that substantiate the role of the immune signaling pathway in the pathophysiology of SSH, particularly emphasizing certain aspects of the neural and renal mechanisms. The salt-induced challenges that prompt the immune system to corroborate pro-hypertensive mechanisms also entail significant alterations in the gut microbiome. Consequently, the trafficking of large amounts of salt through the body not only impairs autonomic and volume-related regulation of the cardiovascular system, but also adversely affects the commensal bacterial community in the gut, which subsequently reverberates in the immune system, contributing to a low-grade, chronic inflammatory state associated with important neural and renal pathophysiological mechanisms. The multiplicity of signaling mechanisms involved and the overlapping effects of aging and sexual dimorphism underscore the complexity of SSH, necessitating novel approaches and further investigation in this field to develop effective management strategies for hypertension. A chart of our working hypothesis is summarized in Figure 1.
